# Association between triglyceride glucose-body mass index and long-term adverse outcomes of heart failure patients with coronary heart disease

**DOI:** 10.1186/s12933-024-02213-2

**Published:** 2024-05-09

**Authors:** Lyu Lyu, Xinhong Wang, Juan Xu, Zhenzhen Liu, Yanru He, Wenjing Zhu, Lin Lin, Benchuan Hao, Hongbin Liu

**Affiliations:** 1 Department of Cardiology, The Second Medical Center, Chinese PLA General Hospital, Beijing, China; 2 Medical School of Chinese PLA, Beijing, China; 3 Department of Cardiology, The Second Affiliated Hospital of Xi’an Jiaotong University, Xi’an, China; 4 Department of General Surgery, Affiliated Xiaoshan Hospital, Hangzhou Normal University, Hangzhou, China

**Keywords:** Triglyceride glucose-body mass index, Heart failure, All-cause mortality, Heart failure rehospitalization, Nonlinear association

## Abstract

**Background:**

The triglyceride glucose-body mass index (TyG-BMI) is recognized as a reliable surrogate for evaluating insulin resistance and an effective predictor of cardiovascular disease. However, the link between TyG-BMI index and adverse outcomes in heart failure (HF) patients remains unclear. This study examines the correlation of the TyG-BMI index with long-term adverse outcomes in HF patients with coronary heart disease (CHD).

**Methods:**

This single-center, prospective cohort study included 823 HF patients with CHD. The TyG-BMI index was calculated as follows: ln [fasting triglyceride (mg/dL) × fasting blood glucose (mg/dL)/2] × BMI. To explore the association between the TyG-BMI index and the occurrences of all-cause mortality and HF rehospitalization, we utilized multivariate Cox regression models and restricted cubic splines with threshold analysis.

**Results:**

Over a follow-up period of 9.4 years, 425 patients died, and 484 were rehospitalized due to HF. Threshold analysis revealed a significant reverse “J”-shaped relationship between the TyG-BMI index and all-cause mortality, indicating a decreased risk of all-cause mortality with higher TyG-BMI index values below 240.0 (adjusted model: HR 0.90, 95% CI 0.86–0.93; Log-likelihood ratio *p* = 0.003). A distinct “U”-shaped nonlinear relationship was observed with HF rehospitalization, with the inflection point at 228.56 (adjusted model: below: HR 0.95, 95% CI 0.91–0.98; above: HR 1.08, 95% CI 1.03–1.13; Log-likelihood ratio *p* < 0.001).

**Conclusions:**

This study reveals a nonlinear association between the TyG-BMI index and both all-cause mortality and HF rehospitalization in HF patients with CHD, positioning the TyG-BMI index as a significant prognostic marker in this population.

**Supplementary Information:**

The online version contains supplementary material available at 10.1186/s12933-024-02213-2.

## Introduction

Heart failure (HF) presents a global challenge, adversely affecting quality of life, causing life-threatening syndromes, and imposing a substantial healthcare burden [1]. Despite earlier prevention efforts and more precise therapies, the prevalence of HF continues to rise, paralleling the aging of the population [2]. Consequently, it becomes imperative to delve deeper into early prognostic biomarkers for adverse outcomes in HF patients.

Insulin resistance (IR), characterized by diminished sensitivity and response to insulin action [3], significantly influences HF. IR precedes cardiac dysfunction in HF by tightly controlling glucose and fatty acid metabolism through insulin signaling in the heart, thereby accelerating HF progression [4]. Its close associations with obesity, diabetes, hypertension, and coronary heart disease (CHD) further implicate IR in HF development. Concurrently, HF patients may exhibit both systemic and cardiac IR [5]. This interplay warrants intensive investigation.

In the current absence of validated methods for accurately assessing IR, the triglyceride-glucose (TyG) index has emerged as an alternative marker [6]. The TyG index, which is based on fasting triglyceride (FTG) and glucose levels [6], has been recognized as a more reliable marker for evaluating IR compared to the euglycemic-hyperinsulinaemic clamp test (The gold-standard for diagnosis of IR) [7] and the homeostasis model assessment-estimated insulin resistance (HOMA-IR) index [8, 9]. Additionally, its convenience and low cost make the TyG index a viable tool for all individuals, regardless of diabetes status, as it eliminates the need for insulin quantification [10]. Recent studies have indicated that TyG-related parameters, including TyG-waist circumference (WC), TyG-waist-to-height ratio (WtHR), and TyG-body mass index (BMI), are particularly valuable in assessing IR [11, 12], with the TyG-BMI index being of notable importance [13]. TyG-BMI index was found to have similar effects with HOMA-IR index for IR assessment in Korean adults [13] and Chinese nondiabetic individuals [11]. Moreover, TyG-BMI index was considered to be an effective index to predict diabetes in the impaired fasting glucose patients than TyG-WC and TyG-WtHR [14]. In addition, considering the close association between IR and obesity [15], the combination of obesity (as defined by BMI) and TyG index can better identify IR than other surrogate markers. Because of the impact of obesity and IR on HF, this combination can also present new insights into the relationship between adverse outcomes and HF.

The prognostic value of the TyG-BMI index in relation to adverse cardiovascular (CV) outcomes, especially all-cause mortality [16, 17],has been corroborated by several studies [16–19]. One cross-sectional study found that TyG-BMI index was negatively correlated with early-onset HF in patients with ST-elevation myocardial infarction who underwent primary percutaneous coronary intervention (PCI) [20]. Dou et al. [21] were the first to report the negative impact of the TyG-BMI index on 360-day all-cause mortality in HF patients. However, there remains a gap in cohort studies exploring the prognostic utility of the TyG-BMI index for long-term adverse outcomes, including all-cause mortality and HF rehospitalization, across all ejection fraction (EF) phenotypes in HF patients. Our study aims to evaluate the association of the TyG-BMI index with long-term adverse outcomes in hospitalized HF patients with CHD.

## Methods

### Study population

This prospective cohort study included consecutive patients with HF recruited from the Department of Cardiology at the Chinese PLA General Hospital in Beijing, China, between October 2010 and September 2014. Eligibility required a diagnosis of HF based on the European Society of Cardiology guidelines [22]. Patients were classified into three categories: HF with reduced ejection fraction (HFrEF), HF with mid-range ejection fraction (HFmrEF), and HF with preserved ejection fraction (HFpEF). The exclusion criteria were: (1) presence of diseases such as moderate or severe valvular heart disease, severe pulmonary hypertension, arrhythmogenic right ventricular dysplasia, congenital heart disease, right ventricular infarction, pericardial disease, or specific cardiomyopathies; (2) a life expectancy of less than one year; (3) absence of key variables. After excluding three patients due to missing key variables and 31 lost to follow-up, a total of 823 patients were ultimately available for long-term analysis, comprising 230 HFrEF, 271 HFmrEF, and 322 HFpEF patients. The missed follow-up rate was 3.6%. The study’s protocol received approval from the ethics committee of the Chinese PLA General Hospital, and all participants provided written informed consent at the initial visit.

### TyG-BMI index

FTG and blood glucose (FBG) levels were obtained from electronic medical records at admission. Blood samples were taken for the measurement of triglyceride (TG) and glucose levels under fasting conditions. The TyG-BMI index was calculated as follows: BMI = weight (kg)/height (m^2^); $\text{T}\text{y}\text{G} \text{i}\text{n}\text{d}\text{e}\text{x} =\text{l}\text{n}\left(\frac{\text{f}\text{a}\text{s}\text{t}\text{i}\text{n}\text{g} \text{t}\text{r}\text{i}\text{g}\text{l}\text{y}\text{c}\text{e}\text{r}\text{i}\text{d}\text{e} \left(\text{m}\text{g}/\text{d}\text{L}\right)\times \text{f}\text{a}\text{s}\text{t}\text{i}\text{n}\text{g} \text{g}\text{l}\text{u}\text{c}\text{o}\text{s}\text{e} (\text{m}\text{g}/\text{d}\text{L})}{2}\right)$ [6]; TyG-BMI index = TyG index×BMI.

### Clinical variables

Clinical characteristics, medical therapy at discharge, and biochemical parameters were collected from medical records. Laboratory indices were determined using standard institutional laboratory measurements at the Chinese PLA General Hospital. All measurements were carried out by personnel blinded to the patients’ baseline characteristics and clinical outcomes.

Atrial fibrillation (AF), chronic obstructive pulmonary disease (COPD), and ischemic stroke during admission were identified based on diagnoses in medical records. CHD was defined as having ≥ 50% stenosis in at least one coronary artery, as determined by coronary arteriography. Hypertension was characterized as having a systolic blood pressure (SBP) ≥ 140 mmHg, diastolic blood pressure (DBP) ≥ 90 mmHg, or being on antihypertensive medication. Diabetes mellitus (DM) was diagnosed based on medical records or positive laboratory test results, specifically Hemoglobin A1c (HbA1c) levels ≥ 6.5%. Chronic kidney disease (CKD) was defined as an estimated glomerular filtration rate (eGFR) of < 60 mL/min/1.73 m^2^.

### Echocardiographic measurements

Two-dimensional color, pulsed-wave, and continuous-wave Doppler echocardiogram (IE33 echocardiography system, Royal Philips Electronics, Amsterdam, The Netherlands) was performed in our study. All subjects underwent echocardiographic measurements by trained and certified sonographers from the Cardiology Department of the Chinese PLA General Hospital according to the guidelines issued by the American Society of Echocardiography [23]. Left atrium dimension (LAD), left ventricular posterior wall thickness (LVPWT), left ventricular end-diastolic dimension (LVEDD), interventricular septum thickness (IVST), left ventricular end-systolic diameter (LVESD), right ventricular diameter (RVD), right atrial diameter (RAD), right ventricular free wall (RVFW), and inferior vena cava (IVC) were measured manually. Left ventricular ejection fraction (LVEF), left ventricular end-diastolic volume (LVEDV), left ventricular end-systolic volume (LVESV), left ventricular fraction shortening (LVFS), early (E) mitral inflow peak/atrial (A) filling peak ratio (E/A), maximum tricuspid regurgitation velocity (TRV max), maximum aortic valve velocity (AVV max), and pulmonary artery pressure (PAP) were measured automatically.

We estimated the left ventricular mass using the formula recommended in the guidelines and then normalized to the left ventricular mass index (LVMI) according to the body surface area (calculated using the formula of Stevenson). The left atrial volume was calculated using the estimated ellipsoid method [24], and then normalized to the left atrial volume index (LAVI) via the above method. LVEF was measured using the modified Simpson’s method in the apical four- and two-chamber views.

### Outcomes and follow-up

The primary outcomes were all-cause mortality and HF rehospitalization, monitored through biennial telephone interviews. All-cause mortality were mortality from all causes, including CV causes (refractory HF, arrhythmia, acute myocardial infarction, cerebrovascular accident) and non-CV death (neoplasia, infection, inflammation, renal failure, multiple organ failure, aortic aneurism, vascular surgery, immune system diseases, and others). These interviews were conducted with participants or their proxies to collect information on any hospitalizations or deaths that occurred during the interval, with the latest follow-up deadline being March 2023. Patients without recorded events by this date were considered right-censored in the analysis.

### Statistical analysis

Continuous variables were described as mean ± standard deviation (SD) or median with interquartile range (IQR), depending on the distribution; categorical variables were presented as counts and percentages. We used the Kruskal–Wallis and χ^2^ tests for descriptive analysis.

In survival analysis, we employed univariate Kaplan–Meier and multivariate Cox regression models to investigate the relationship between the TyG-BMI index and long-term adverse outcomes. We compared Kaplan–Meier curves using the log-rank test. The TyG-BMI index was treated as a categorical variable in the multivariate Cox model. This model adjusted for factors including age, gender, smoking, SBP, heart rate, DM, hypertension, history of myocardial infarction, prior PCI/coronary artery bypass grafting (CABG), stroke, CKD, anemia, COPD, AF, LVEF, and medications such as statins, beta blockers, angiotensin-converting enzyme inhibitors (ACE-I)/angiotensin II receptor blockers (ARBs), diuretics, spironolactone, digoxin, and calcium channel blockers. Additional adjustments were made for creatinine, total cholesterol (TC), low-density lipoprotein cholesterol (LDL-C), N-terminal pro-brain natriuretic peptide (NT-proBNP), and high-sensitivity cardiac troponin T (hs-TnT). Variables like age, SBP, heart rate, LVEF, creatinine, TC, LDL-C, NT-proBNP, and hs-TnT were log-transformed in this model. We assessed the nonlinear relationship between the TyG-BMI index (as a continuous variable) and adverse outcomes using restricted cubic splines (RCS). RCS defined a threshold value for a segmented fit of outcomes in the Cox regression model.

All statistical analyses were performed using R version 4.1.2 (The R Project for Statistical Computing, Vienna, Austria). We considered a two-tailed *p*-value of less than 0.05 as statistically significant.

## Results

### Baseline characteristics

Table 1 displays the baseline characteristics of the 823 HF patients with CHD, categorized according to their TyG-BMI index levels. The median age was 68.0 years, with an interquartile range of 56.0–77.0 years. The TyG-BMI index varied from 114.0 to 386.68, with an average value of 222.5 and a standard deviation of 40.5. Patients with higher TyG-BMI index levels were typically younger and demonstrated elevated BMI, DBP, glucose, HbA1c, TC, TG, and TyG index values. A greater prevalence of male patients, current smokers, and DM was noted in this subgroup. In contrast, these patients had reduced high-density lipoprotein cholesterol (HDL-C) and NT-proBNP levels, along with a lower incidence of COPD. Additionally, notable statistical differences were observed in echocardiographic measurements among the three groups. These measurements included LAD, LVPWT, LVEDD, IVST, LVESD, LVEDV, LVESV, RVFW, IVC, TRV max, and PAP.

**Table 1:** Baseline characteristics of study patients according to status of TyG-BMI index

Baseline characteristics	Total (n = 823)	Low TyG-BMI index (n = 274)	Medium TyG-BMI index (n = 275)	High TyG-BMI index (n = 274)	p-value
Age, median (IQR), years	68.0 (56.0–77.0)	73.0 (65.0–81.0)	68.0 (56.0–76.0)	62.0 (52.0–73.0)	< 0.001
Male, n (%)	578 (70.2%)	178 (65.0%)	197 (69.0%)	203 (74.1%)	0.012
Current smokers, n (%)	218 (26.5%)	60 (21.9%)	67 (24.4%)	91 (33.2%)	0.003
Body mass index, mean (SD), kg/m²	24.9 ± 3.6	21.9 ± 2.8	25.1 ± 2.1	27.9 ± 3.1	< 0.001
Systolic blood pressure, median (IQR), mm Hg	131.0 (118.0–147.0)	129.0 (114.0–145.0)	130.0 (120.0–147.0)	134.0 (120.0–150.0)	0.063
Diastolic blood pressure, median (IQR), mm Hg	72.0 (64.0–80.0)	70.0 (61.0–80.0)	72.0 (65.0–80.0)	75.0 (68.0–84.0)	< 0.001
Heart rate, median (IQR), bpm	74.0 (66.0–84.0)	73.0 (65.0–84.0)	73.0 (67.0–82.0)	75.0 (68.0–84.0)	0.488
NYHA-FC, n (%)					0.389
I/II	522 (63.4%)	169 (61.7%)	188 (68.4%)	165 (60.2%)	
III	212 (25.8%)	72 (26.3%)	64 (23.3%)	76 (27.6%)	
IV	89 (10.8%)	37 (13.5%)	24 (8.7%)	28 (10.2%)	
*Medical history*					
Diabetes mellitus, n (%)	328 (39.9%)	79 (28.8%)	104 (37.8%)	145 (52.9%)	< 0.001
Hypertension, n (%)	556 (67.6%)	181 (66.1%)	183 (66.5%)	192 (70.1%)	0.253
Previous myocardial infarction, n (%)	272 (33.1%)	92 (33.6%)	95 (34.5%)	85 (31.0%)	0.799
Previous PCI/CABG, n (%)	343 (41.7%)	107 (39.1%)	123 (44.7%)	113 (41.2%)	0.346
Stroke, n (%)	109 (13.2%)	46 (16.8%)	31 (11.3%)	32 (11.7%)	0.133
Chronic kidney disease, n (%)	131 (15.9%)	47 (17.2%)	45 (16.4%)	39 (14.2%)	0.727
Anemia, n (%)	63 (7.7%)	26 (9.5%)	20 (7.3%)	17 (6.2%)	0.391
Chronic obstructive pulmonary disease, n (%)	83 (10.1%)	41 (15.0%)	24 (8.7%)	18 (6.6%)	0.005
Atrial fibrillation, n (%)	122 (14.8%)	48 (17.5%)	39 (14.2%)	35 (12.8%)	0.347
*Echocardiography*					
Left ventricular ejection fraction, median (IQR), %	46.0 (40.0–55.0)	46.0 (38.0–56.0)	45.0 (40.0–55.0)	45.0 (41.0–55.0)	0.922
LAD, median (IQR), mm	39.0 (35.0–43.0)	38.0 (34.0–41.0)	39.0 (35.0–42.0)	40.0 (36.0–43.0)	< 0.001
LVPWT, median (IQR), mm	10.0 (10.0–11.0)	10.0 (9.0–11.0)	11.0 (10.0–11.0)	11.0 (10.0–11.0)	< 0.001
LVEDD, median (IQR), mm	49.0 (45.0–54.0)	48.0 (44.0–53.0)	48.0 (45.0–53.0)	51.0 (46.0–57.0)	0.001
IVST, median (IQR), mm	11.0 (10.0–12.0)	11.0 (10.0–12.0)	11.0 (10.0–12.0)	11.0 (10.0–12.0)	0.011
LVMI, median (IQR), g/m^2^	108.2 (92.7–130.0)	109.2 (94.4–130.9)	108.0 (91.9–129.3)	107.6 (92.5–129.8)	0.589
LAVI, median (IQR), mL/m^2^	46.7 (36.2–60.0)	47.1 (37.0–63.1)	46.6 (35.5–60.3)	46.1 (37.1–57.8)	0.510
LVESD, median (IQR), mm	36.0 (32.0–41.0)	36.0 (31.0–41.0)	35.0 (31.0–40.8)	37.0 (32.0–43.0)	0.031
LVEDV, median (IQR), mm	112.0 (90.0–141.0)	108.0 (85.0–139.3)	109.5 (88.8–133.3)	120.0 (97.0–153.0)	0.002
LVESV, median (IQR), mm	58.0 (41.0–82.0)	56.0 (38.0–84.0)	55.0 (41.0–78.3)	61.5 (45.3–85.0)	0.028
LVFS, median (IQR), mm	26.0 (21.0–30.0)	27.0 (20.0–30.0)	26.0 (21.0–30.0)	26.0 (21.0–30.0)	0.941
RVD, median (IQR), mm	35.0 (32.0–38.0)	34.0 (31.0–37.0)	35.0 (32.0–38.0)	35.0 (32.0–38.0)	0.233
RAD, median (IQR), mm	35.0 (32.0–38.0)	34.0 (31.0–38.0)	35.0 (32.0–38.0)	34.0 (32.0–37.0)	0.870
RVFW, median (IQR), mm	6.0 (5.0–6.0)	5.0 (5.0–6.0)	6.0 (5.0–6.0)	6.0 (5.0–6.0)	0.001
IVC, median (IQR), mm	15.0 (14.0–17.0)	15.0 (14.0–17.0)	15.0 (14.0–16.0)	15.0 (14.0–17.0)	0.013
E/A, median (IQR)	0.8 (0.6–1.3)	0.8 (0.6–1.3)	0.8 (0.6–1.3)	0.8 (0.6–1.3)	0.388
TRV max, median (IQR), m/s	2.3 (2.1–2.7)	2.4 (2.1–2.9)	2.3 (2.1–2.6)	2.3 (2.1–2.6)	0.005
AVV max, median (IQR), m/s	1.2 (1.0–1.4)	1.2 (1.0–1.5)	1.2 (1.0–1.4)	1.2 (1.0–1.4)	0.938
PAP, median (IQR), mm Hg	29.0 (23.0–36.0)	30.0 (25.0–39.0)	29.0 (23.0–35.0)	28.0 (21.0–34.0)	< 0.001
*Medication*					
Statin, n (%)	749 (91.1%)	247 (90.1%)	252 (91.6%)	250 (91.2%)	0.228
Beta blocker, n (%)	635 (77.3%)	204 (74.5%)	215 (78.2%)	216 (78.8%)	0.141
ACE-I/ARB, n (%)	408 (49.6%)	124 (45.3%)	143 (52.0%)	141 (51.5%)	0.119
Diuretic, n (%)	309 (37.5%)	111 (40.5%)	101 (36.7%)	97 (35.4%)	0.597
Spironolactone, n (%)	349 (42.4%)	127 (46.4%)	107 (38.9%)	115 (42.0%)	0.255
Digoxin, n (%)	130 (15.8%)	52 (19.0%)	36 (13.1%)	42 (15.3%)	0.181
Calcium channel blocker, n (%)	234 (28.5%)	74 (27.0%)	78 (28.4%)	82 (29.9%)	0.604
*Laboratory indicators*					
Creatinine, median (IQR), mg/dL	0.9 (0.8–1.2)	0.9 (0.8–1.2)	0.9 (0.8–1.1)	0.9 (0.8–1.2)	0.801
Glucose, median (IQR), mmol/L	6.4 (5.1–8.6)	6.3 (5.2–8.5)	6.3 (5.2–8.5)	6.4 (5.1–8.8)	< 0.001
HbA1c, median (IQR), %	6.2 (5.8–7.2)	6.0 (5.6–6.6)	6.1 (5.8–7.0)	6.8 (6.0–7.9)	< 0.001
HDL-C, median (IQR), mmol/L	1.0 (0.8–1.2)	1.1 (0.9–1.3)	1.0 (0.8–1.2)	0.9 (0.8–1.1)	< 0.001
LDL-C, median (IQR), mmol/L	2.2 (1.7–2.9)	2.2 (1.7–2.7)	2.2 (1.8–2.8)	2.3 (1.7–3.1)	0.092
Total cholesterol, median (IQR), mmol/L	3.8 (3.2–4.6)	3.8 (3.2–4.4)	3.7 (3.3–4.5)	3.9 (3.3–4.8)	0.037
Triglycerides, median (IQR), mmol/L	1.3 (0.9–1.8)	0.9 (0.7–1.4)	1.2 (1.0–1.7)	1.7 (1.3–2.4)	< 0.001
NT-proBNP, median (IQR), pg/mL	1,063.0 (354.5–1,896.5)	1,503.5 (489.3–4,692.3)	1,049.0 (335.8–2,866.3)	865.0 (268.0–2,463.0)	< 0.001
hs-TnT, median (IQR), ng/L	0.03 (0.01–0.11)	0.03 (0.01–0.11)	0.02 (0.01–0.11)	0.03 (0.01–0.16)	0.253
TyG index, mean (SD)	8.9 ± 0.7	8.5 ± 0.6	8.8 ± 0.5	9.3 ± 0.7	< 0.001
TyG-BMI index, mean (SD)	222.5 ± 40.5	180.6 ± 20.5	221.3 ± 8.3	267.1 ± 26.4	< 0.001

TyG-BMI index, triglyceride glucose-body mass index; IQR, Inter-quartile range; NYHA-FC, New York Heart Association functional class; PCI, Percutaneous coronary intervention; CABG, Coronary artery bypass grafting; LAD, Left atrium dimension; LVPWT, Left ventricular posterior wall thickness; LVEDD, Left ventricular end-diastolic dimension; IVST, Interventricular septum thickness; LVMI, Left ventricular mass index; LAVI, Left atrial volume index; LVESD, Left ventricular end systolic diameter; LVEDV, Left ventricular end-diastolic volume; LVESV, Left ventricular end-systolic volume; LVFS, Left ventricular fraction shortening; RVD, Right ventricular diameter; RAD, Right atrial diameter; RVFW, Right ventricular free wall; IVC, Inferior vena cava; E/A, early (E) mitral inflow peak/atrial (A) filling peak ratio; TRV, Tricuspid regurgitation velocity; AVV, Aortic valve velocity; PAP, Pulmonary artery pressure; IQR, Inter-quartile range; ACE-I, Angiotensin-converting enzyme inhibitor; ARB, Angiotensin II receptor blocker; HbA1c, Hemoglobin A1c; HDL-C, High-density lipoprotein cholesterol; LDL-C, Low-density lipoprotein cholesterol; NT-proBNP, N-terminal pro-brain natriuretic peptide, hs-TnT, high-sensitivity cardiac troponin T; TyG, triglyceride-glucose

### Association between the TyG-BMI index and adverse outcomes

**Fig. 1:**
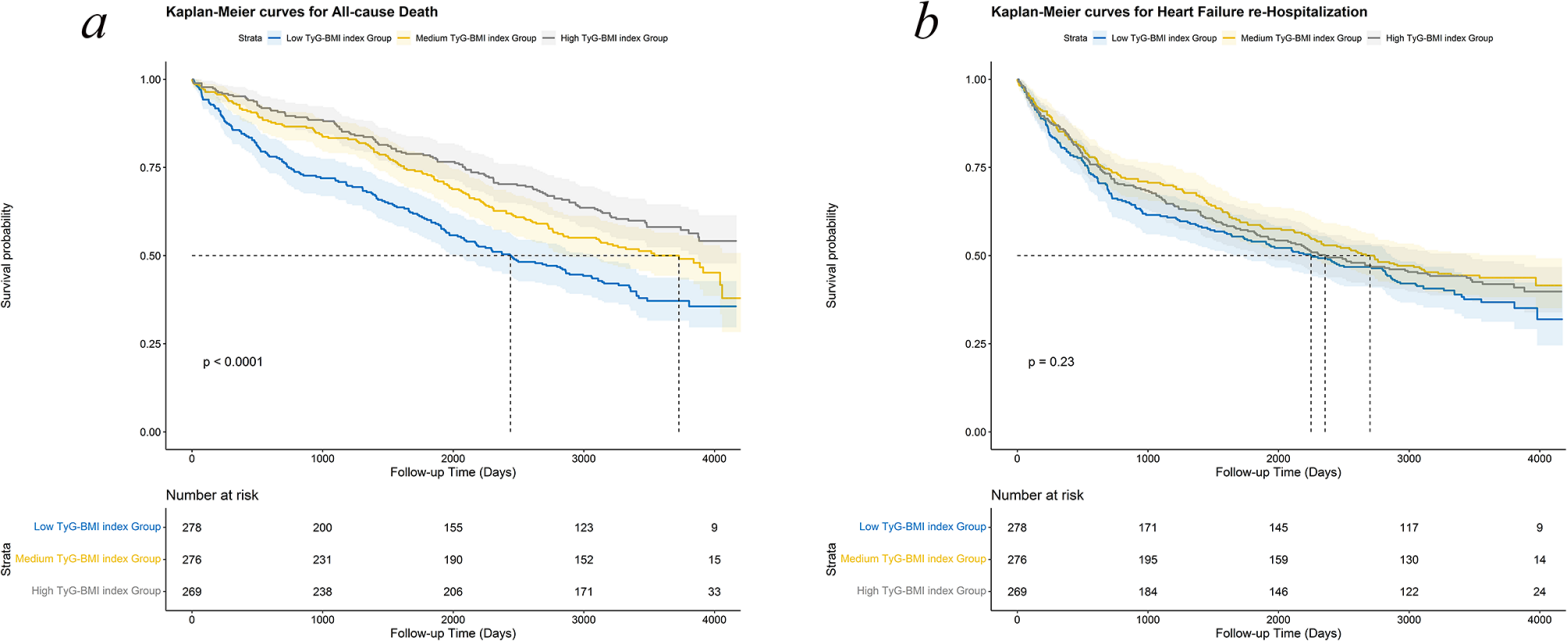
Kaplan–Meier survival curves for adverse outcomes in all heart failure patients. Kaplan–Meier survival curves for (**a**) all-cause mortality and (**b**) HF rehospitalizition HF, heart failure

The median follow-up period was 9.4 years (range 8.7–10.5 years). During this time, 425 patients experienced all-cause mortality, and 484 patients underwent HF rehospitalization. Kaplan–Meier survival analysis, depicted in Fig. 1, illustrated the cumulative survival probabilities for both all-cause mortality and HF rehospitalization across three groups, categorized by their TyG-BMI index levels. The group with a higher TyG-BMI index demonstrated a significantly lower rate of all-cause mortality compared to those with a lower TyG-BMI index (*p* < 0.001). However, the rates of HF rehospitalization did not significantly differ among the groups (*p* = 0.23; Fig. 1).

**Fig. 2:**
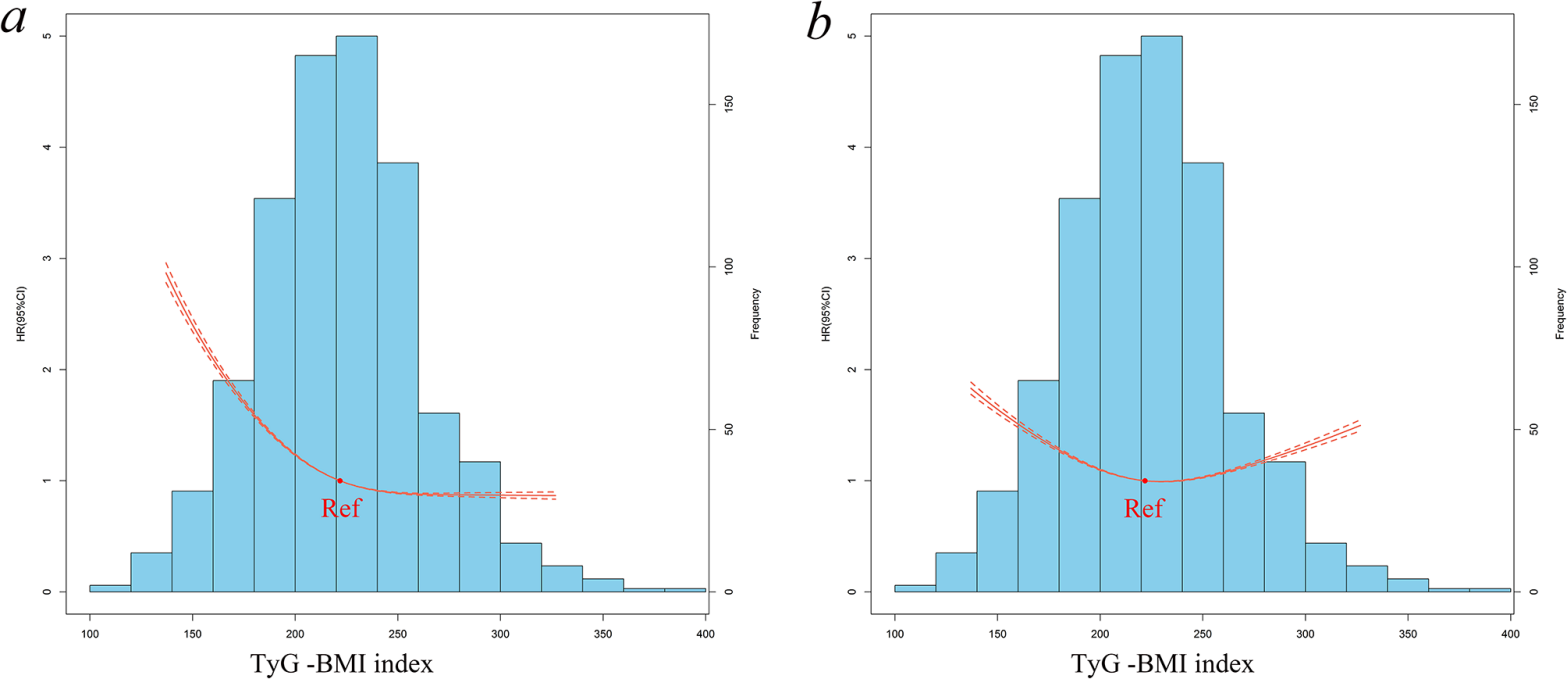
Association between TyG-BMI index and adverse outcomes using a restricted cubic spline (RCS) regression model and histogram. (**a**) all-cause mortality and (**b**) HF rehospitalizition. The model was adjusted for age, gender, smoking, SBP, heart rate, diabetes mellitus, hypertension, previous myocardial infarction, previous PCI/CABG, stroke, chronic kidney disease, anemia, COPD, atrial fibrillation, LVEF, statin, beta blocker, ACE-I/ARB, diuretic, spironolactone, digoxin, calcium channel blocker, creatinine, TC, LDL-C, NT-proBNP, hs-TnT. HR, hazard ratio; CI, confidence interval; TyG-BMI index, triglyceride glucose-body mass index; SBP, systolic blood pressure; PCI, percutaneous coronary intervention; CABG, coronary artery bypass grafting; COPD, chronic obstructive pulmonary disease; LVEF, left ventricular ejection fraction; ACE-I, angiotensin-converting enzyme inhibitor; ARB, angiotensin II receptor blocker; TC, total cholesterol; LDL-C, low-density lipoprotein cholesterol; NT-proBNP, N-terminal pro-brain natriuretic peptide, hs-TnT, high-sensitivity cardiac troponin T

When the TyG-BMI index was included as a categorical variable in the multivariable Cox regression model, it facilitated the evaluation of its association with adverse outcomes. The results indicated potential nonlinear relationships between the TyG-BMI index and both all-cause mortality and HF rehospitalization (Table 2; Fig. 2).

**Table 2:** Association between TyG-BMI index and adverse outcomes in heart failure patients

Variables	Total incidence	Tertiles of TyG-BMI index			p for trend
		Low TyG-BMI index (114.04–205.79)	Medium TyG-BMI index (205.80–235.52)	High TyG-BMI index (235.78–386.68)	
All-cause mortality					
Events/ sample size	425/ 823	167/ 274	140/ 275	118/ 274	
Incidence per 1,000 PYs (95% CI)	233.46 (212.29–256.74)	268.28 (230.53–312.22)	221.63 (187.80–261.57)	208.37 (173.97–249.57)	
Crude HR (95% CI)	——	1.43 (1.14–1.78)	Ref.	0.76 (0.60–0.98)	< 0.001
Model 1^a^: adjusted HR (95% CI)	——	1.14 (0.91–1.43)	Ref	0.91 (0.71–1.17)	0.196
Model 2^b^: adjusted HR (95% CI)	——	1.00 (0.79–1.26)	Ref.	0.93 (0.72–1.20)	0.380
**Heart failure rehospitalization**					
Events/ sample size	484/ 823	173/ 274	155/ 275	156/ 274	
Incidence per 1,000 PYs (95% CI)	316.65 (289.66–346.15)	322.94 (278.23–374.83)	304.77 (260.37–356.73)	322.17 (275.38–376.91)	
Crude HR (95% CI)	——	1.20 (0.97–1.49)	Ref.	1.06 (0.85–1.32)	0.237
Model 1^a^: adjusted HR (95% CI)	——	1.16 (0.94–1.45)	Ref.	1.10 (1.02–1.37)	0.175
Model 2^b^: adjusted HR (95% CI)	——	1.12 (0.90–1.40)	Ref.	1.14 (0.91–1.43)	0.254

^a^ Model 1 adjusted for age, gender

^b^ Model 2 adjusted for age, gender, smoking, SBP, heart rate, diabetes mellitus, hypertension, previous myocardial infarction, previous PCI/CABG, stroke, chronic kidney disease, anemia, COPD, atrial fibrillation, LVEF, statin, beta blocker, ACE-I/ARB, diuretic, spironolactone, digoxin, calcium channel blocker, creatinine, TC, LDL-C, NT-proBNP, hs-TnT

PY, person-year; HR, hazard ratio; CI, confidence interval; TyG-BMI index, triglyceride glucose-body mass index; SBP, systolic blood pressure; PCI, percutaneous coronary intervention; CABG, coronary artery bypass grafting; COPD, chronic obstructive pulmonary disease; LVEF, left ventricular ejection fraction; ACE-I, angiotensin-converting enzyme inhibitor; ARB, angiotensin II receptor blocker; TC, total cholesterol; LDL-C, low-density lipoprotein cholesterol; NT-proBNP, N-terminal pro-brain natriuretic peptide, hs-TnT, high-sensitivity cardiac troponin T

### Detection of nonlinear association of the TyG-BMI index with adverse outcomes

**Fig. 3:**
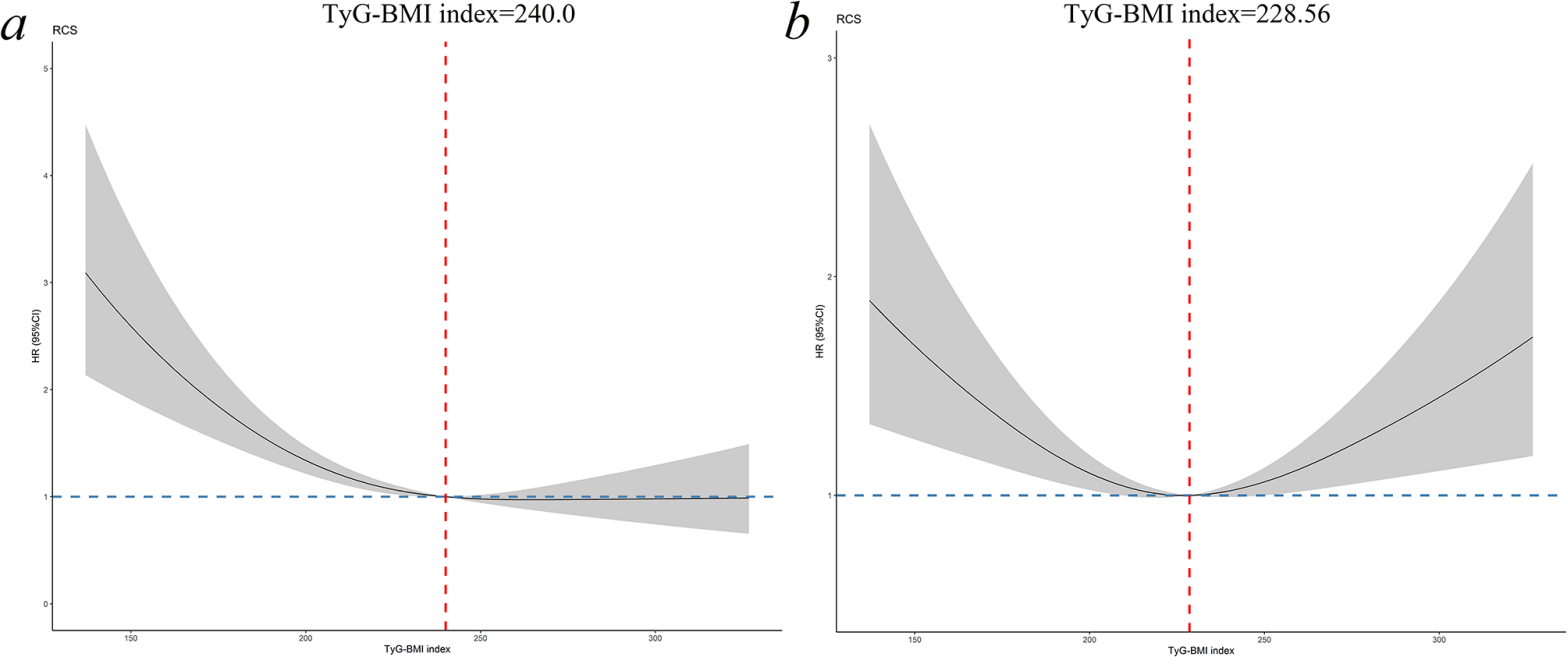
The nonlinear association of TyG-BMI index with adverse outcomes in the fully adjusted model. The nonlinear association of TyG-BMI index (as a continuous variable) with (**a**) all-cause mortality and (**b**) HF rehospitalizition. Spline curves were adjusted for age, gender, smoking, SBP, heart rate, diabetes mellitus, hypertension, previous myocardial infarction, previous PCI/CABG, stroke, chronic kidney disease, anemia, COPD, atrial fibrillation, LVEF, statin, beta blocker, ACE-I/ARB, diuretic, spironolactone, digoxin, calcium channel blocker, creatinine, TC, LDL-C, NT-proBNP, hs-TnT. HR, hazard ratio; CI, confidence interval; TyG-BMI index, triglyceride glucose-body mass index; HF, heart failure; SBP, systolic blood pressure; PCI, percutaneous coronary intervention; CABG, coronary artery bypass grafting; COPD, chronic obstructive pulmonary disease; LVEF, left ventricular ejection fraction; ACE-I, angiotensin-converting enzyme inhibitor; ARB, angiotensin II receptor blocker; TC, total cholesterol; LDL-C, low-density lipoprotein cholesterol; NT-proBNP, N-terminal pro-brain natriuretic peptide, hs-TnT, high-sensitivity cardiac troponin T

Cox proportional hazards regression models using RCS were employed to investigate the nonlinear association between the TyG-BMI index and adverse outcomes. The analysis revealed a significant reverse “J”-shaped relationship between the TyG-BMI index and all-cause mortality in the fully adjusted model (*p* for nonlinearity = 0.004; Fig. 3a). Additionally, the TyG-BMI index demonstrated a significant “U”-shaped nonlinear association with HF rehospitalization (*p* for nonlinearity = 0.002; Fig. 3b).

Two-piecewise Cox proportional hazards regression models, with threshold-specific associations identified by RCS with three knots, were used. The results indicated a decrease in the risk of all-cause mortality with an increase in the TyG-BMI index up to a threshold of 240.0. Beyond this threshold, the relationship between the TyG-BMI index and all-cause mortality was not significant (TyG-BMI index < 240.0: per unit increase, HR 0.90, 95% CI 0.86–0.93; TyG-BMI index > 240.0: per unit increase, HR 1.03, 95% CI 0.97–1.10; Fig. 3a; Table 3). The inflection point for HF rehospitalization was identified at 228.56. An increased TyG-BMI index below this inflection point was associated with a decreased risk of HF rehospitalization. In contrast, above the inflection point, higher TyG-BMI index levels were positively associated with an increased risk of HF rehospitalization (TyG-BMI index < 228.56: per unit increase, HR 0.95, 95% CI 0.91–0.98; TyG-BMI index > 228.56: per unit increase, HR 1.08, 95% CI 1.03–1.13; Fig. 3b; Table 3).

**Table 3:** Threshold effect analysis of TyG-BMI index on all-cause mortality and heart failure rehospitalization

	Crude HR (95% CI)	Adjusted HR^a^ (95% CI)
All-cause mortality		
Total	0.93 (0.90–0.96)	0.94 (0.91–0.97)
Fitting by two-piecewise Cox regression model		
Inflection point	252.44	240.00
TyG-BMI index < inflection point (per unit)	0.89 (0.86–0.92)	0.90 (0.86–0.93)
TyG-BMI index > inflection point (per unit)	1.05 (0.99–1.12)	1.03 (0.97–1.10)
*p* for Log-likelihood ratio	< 0.001	0.003
**Heart failure rehospitalization**		
Total	0.99 (0.97–1.02)	1.01 (0.98–1.03)
Fitting by two-piecewise Cox regression model		
Inflection point	244.56	228.56
TyG-BMI index < inflection point (per unit)	0.96 (0.93–0.99)	0.95 (0.91–0.98)
TyG-BMI index > inflection point (per unit)	1.17 (1.09–1.25)	1.08 (1.03–1.13)
*p* for Log-likelihood ratio	< 0.001	< 0.001

^a^ Adjusted for age, gender, smoking, SBP, heart rate, diabetes mellitus, hypertension, previous myocardial infarction, previous PCI/CABG, stroke, chronic kidney disease, anemia, COPD, atrial fibrillation, LVEF, statin, beta blocker, ACE-I/ARB, diuretic, spironolactone, digoxin, calcium channel blocker, creatinine, TC, LDL-C, NT-proBNP, hs-TnT

HR, hazard ratio; CI, confidence interval; TyG-BMI index, triglyceride glucose-body mass index; SBP, systolic blood pressure; PCI, percutaneous coronary intervention; CABG, coronary artery bypass grafting; COPD, chronic obstructive pulmonary disease; LVEF, left ventricular ejection fraction; ACE-I, angiotensin-converting enzyme inhibitor; ARB, angiotensin II receptor blocker; TC, total cholesterol; LDL-C, low-density lipoprotein cholesterol; NT-proBNP, N-terminal pro-brain natriuretic peptide, hs-TnT, high-sensitivity cardiac troponin T

### Subgroup analysis of the relationship between the TyG-BMI index and adverse outcomes

Our investigation focused on the nonlinear relationship between the TyG-BMI index and adverse outcomes in non-diabetic patients using the adjusted model. We identified a reverse “J”-shaped association (nonlinear *p* < 0.01; *p* for Log-likelihood ratio < 0.001; Figure S1c) and a “U”-shaped association (nonlinear *p* = 0.003; *p* for Log-likelihood ratio < 0.001; Figure S1d) independently for the TyG-BMI index with all-cause mortality and HF rehospitalization, respectively, in non-diabetic individuals. In contrast, among diabetic patients, a linear relationship was noted, showing increased TyG-BMI index correlating with decreased risk of all-cause mortality (nonlinear *p* = 0.452; Figure S1a). Nevertheless, in the diabetic subgroup, the TyG-BMI index did not demonstrate a significant association with HF rehospitalization (nonlinear *p* = 0.318; Figure S1b).

Furthermore, the appendix contains figures representing the separate relationships of the TyG index, BMI, FTG, and FBG with all-cause mortality and HF rehospitalization (Additional file, Figure S2, S3, S4, S5).

## Discussion

In this study, we observed a significant nonlinear relationship between the baseline TyG-BMI index and adverse outcomes, which include all-cause mortality and HF rehospitalization, over an extended period in HF patients across all EF phenotypes with CHD. The threshold analysis identified a distinct inflection point in the TyG-BMI index’s association with adverse outcomes. Notably, the TyG-BMI index exhibited a significant reverse “J”-shaped relationship with all-cause mortality and a “U”-shaped association with HF rehospitalization. Our findings indicate that the TyG-BMI index is an independent and notable predictor of adverse outcomes in HF patients with CHD.

### TyG-BMI index and adverse outcomes in HF patients

Before this study, a single cohort study using the MIMIC-IV database first confirmed a close correlation between the TyG-BMI index and all-cause mortality in HF patients [21]. This study observed that higher levels of the TyG-BMI index were significantly associated with a decreased risk of 360-day all-cause mortality [21]. In our research, which included HF patients across all phenotypes, we similarly found that an elevated TyG-BMI index was linked to a lower risk of all-cause mortality, particularly when the TyG-BMI index was below 240.0 in the adjusted model over a longer follow-up period. Additionally, our study identified a “U”-shaped association between the TyG-BMI index and HF rehospitalization, with an inflection point of 228.56 for HF rehospitalization (Table 3). This finding was reported for the first time in our study. Furthermore, the nonlinear association between the TyG-BMI index and adverse outcomes persisted in the non-diabetic subgroup, where the TyG-BMI index showed a “U”-shaped association with HF rehospitalization and a reverse “J”-shaped relationship with all-cause mortality. Conversely, in diabetic patients, a transition to a linear association between the TyG-BMI index and all-cause mortality was observed, suggesting that an increase in the TyG-BMI index was associated with a decreased risk of all-cause mortality. This finding aligns with previous research [21]. However, the association with HF rehospitalization in the diabetic subgroup was not significant (Figure S1). To date, no other studies have reported on the relationship between the TyG-BMI index and HF rehospitalization in diabetic HF patients. Further research is required to elucidate these findings.

### Increased levels of TyG-BMI index and its potential mechanisms for HF rehospitalization risk

IR is closely associated with HF, independent of diabetes and other relevant metabolic diseases, for several reasons [25, 26]. Firstly, hyperinsulinemia leads to sodium retention [27], which increases myocardial mass and causes subclinical myocardial dysfunction, ultimately reducing cardiac output [28]. Secondly, hyperinsulinemia contributes to sympathetic nervous system activation [29], impairing cardiac innervations and exacerbating cardiac function decline in HF [30]. Additionally, IR is linked to an enhanced pressor effect of angiotensin II, promoting cardiomyocyte hypertrophy and collagen production [31, 32], leading to abnormal cardiac remodeling and dysfunction [33]. Conversely, HF may induce a state of IR [34] and hasten IR progression. Reduced arterial filling due to HF triggers norepinephrine release [35], impairing insulin sensitivity and glucose tolerance [36], thereby leading to subsequent IR. Moreover, HF alters glucose uptake in cardiac cells, favoring free fatty acid use, which results in metabolic dysfunction and subsequent IR [37, 38]. The biological mechanisms underlying rehospitalization in HF patients associated with an elevated TyG-BMI index, as a reliable surrogate for IR, can be elucidated by this interrelated cycle.

### Lower levels of TyG-BMI index and its potential mechanisms for adverse outcomes

The observed inverse relationship between the TyG-BMI index and all-cause mortality in HF patients was presumably effected simultaneously by BMI and IR. IR is closely associated with obesity [39, 40]. More than 70% of obese population are IR [41], and overweight or obese individuals may better endure the impact of IR than low/normal weight individuals [42]. Obesity, a key component and common partner of IR, is protective in patients with established HF. Such phenomenon, known as “obesity paradox” (OP) [43], accounts for the negative relationship of TyG-BMI index with all-cause death in HF patients. This trend has been consistently reported across various cohort studies [44–48]. The OP may be explained by several mechanisms. First, chronic HF is often accompanied by a chronic catabolic state, leading to the loss of both fat and lean mass, which in turn results in a poorer prognosis [49]. A higher BMI might indicate better protection and sufficient physiological reserves to combat malnutrition-related inflammation [49]. Second, HF is linked to an anabolic overdrive that offers protection against potential adverse outcomes [26]. Additionally, anti-inflammatory adipose tissue produces soluble tumor necrosis factor-α receptors, mitigating the harmful proapoptotic effects of tumor necrosis factor-α on the myocardium, thus providing a cardioprotective effect [50]. Obesity is also associated with elevated levels of lipoproteins, which bind and neutralize lipopolysaccharides, agents responsible for the release of inflammatory cytokines [50]. Furthermore, lower NT-proBNP levels are observed in obese patients, indicative of a more favorable hemodynamic status characterized by increased blood volume and higher BP. This could potentially allow these patients to tolerate higher doses of cardioprotective medications [51]. Our study demonstrated a similar trend in NT-proBNP levels across different TyG-BMI index categories (Table 1).

IR itself may also benefit HF patients in all-cause death. Obese individuals with the lowest HOMA-IR values have the highest risk of CV mortality [52]. Considering the potential benefits of IR in obese people, the absence of IR in these individuals may deactivate the necessary internal self-defense mechanisms against obesity [52]. Moreover, the reduction of IR may result in potential organ damage [53], and the lack of IR defense mechanisms has possibly been implicated in an increased CV death risk [54]. Like various longevity-promoting interventions, impaired insulin/insulin-like growth factor-1 signaling can serve as molecular signals to exert downstream effects to ultimately induce endogenous defense mechanisms like elevated antioxidant defense capacities culminating in increased stress resistance and longevity [55]. Therefore, while IR is associated with harmful effects, it is also an evolutionary protective mechanism against some dangerous threat to life homeostasis [53]. This protective mechanism of IR may explain the too low TyG-BMI index levels were associated with the lowest survival rates and an increased risk of HF rehospitalization.

### Better predictive performance of TyG-BMI index compared to separate indicators

Previous research indicated that low FTG levels was a predictive biomarker for cardiac mortality in HF patients [56], and extremely low levels of TG are associated with adverse health outcomes [57]. Figure S4 represented a negative correlation between FTG and all-cause mortality and HF rehospitalization in the adjusted model. Similarly, hypoglycemia may trigger adverse CV events [58]. In this study, significant linear association was found between FBG and rehospitalization of HF, while no association was observed between FBG and all-cause death (Additional file, Figure S5). Using FBG or FTG levels alone to predict adverse outcomes may not be sensitive and comprehensive enough. The TyG index, a combination of these two indicators, still has limited predictive performance in our study. Some cohort studies have shown a nonlinear relationship between the TyG index and the risk of HF rehospitalization [59, 60]. Our study observed a similar trend (Additional file, Figure S3b). Prior research has indicated that a higher TyG index independently increases the risk of all-cause mortality in HF patients [59, 61, 62]. However, in this study, no significant association was found between the TyG index and all-cause mortality (Additional file, Figure S3a). Earlier studies have suggested that a lower BMI is not associated with rehospitalization in HF patients [63], while an increasing BMI is significantly linked to a higher risk of HF hospitalization, despite a reduced mortality risk associated with higher BMI levels [64, 65]. The “obesity paradox” does not seem to influence the risk of HF rehospitalization among HF patients. Our study also found a similar pattern in the relationship between BMI and adverse outcomes (Additional file, Figure S2). Overall, considering the trends of FTG, FBG, and BMI, the predictive value of TyG-BMI index on adverse outcomes risk has been influenced by the combined effect of all three factors. TyG-BMI index may be proved to better reveal their interactions and synergistic effects and thus more accurately predict adverse outcomes risk of HF patients in our study, demonstrating significant reverse “J”-shaped relationship with all-cause mortality and a “U”-shaped association with HF rehospitalization.

Our research demonstrated a substantial nonlinear relationship between the TyG-BMI index and long-term adverse outcomes in patients with HF, offering valuable insights for risk stratification, prognosis assessment, and therapeutic guidance in this population. The findings suggest that the TyG-BMI index is an independent predictor of adverse outcomes in HF. Integrating the TyG-BMI index with established risk factors could enhance the precision of risk stratification in HF patients. Furthermore, the TyG-BMI index, as a predictive biomarker for adverse outcomes, can contribute to the effective management of HF patients, allowing for more targeted medication strategies based on different TyG-BMI categories.

### Study strengths and limitations

A key contribution of our study is the novel identification of nonlinear associations between the TyG-BMI index and long-term adverse outcomes in HF patients across all EF phenotypes associated with CHD. Notably, our study is the first to report a “U”-shaped correlation between the TyG-BMI index and rehospitalization in HF patients. However, our study has limitations. Firstly, aside from the TyG-BMI index, TyG-WC and TyG-WtHR are other commonly used metrics for assessing IR. Due to the lack of relevant data in our database, we were unable to compare the TyG-BMI index with these metrics. Secondly, although we adjusted for various confounding factors, there were other confounding factors, such as demographic characters (educational and socioeconomic status), lifestyle variables (frequency of exercise, work and life pressure, and mental health), nutritional levels (quantity or type of adiposity, and dietary habits), or comorbidities that might impact the observed outcomes. Thirdly, our study relied on a single baseline blood sample to gather information on TyG-BMI index, which may alter over follow-up owing to the participants’ lifestyles and medications. Therefore, we could not assess the impact on all-cause mortality and HF rehospitalization over time. Fourthly, nearly 40% of the patients were under antidiabetic treatment and a few subjects took fibrates, which inevitably affected levels of FTG or FBG included in TyG-BMI index calculation and the stability of our results. Moreover, this single-center study, conducted in China, includes a moderate sample size of HF patients with CHD. This could introduce potential selection bias, and the findings might not be universally applicable to other HF populations.

## Conclusion

This study reveals a significant nonlinear association between the TyG-BMI index and both all-cause mortality and HF rehospitalization among HF patients across various EF phenotypes with CHD during long-term follow-up. The TyG-BMI index proves to be a valuable biomarker for predicting the risk of adverse outcomes in HF patients, and its assessment could refine prognosis evaluation for these patients.

### Electronic supplementary material

Below is the link to the electronic supplementary material.

Supplementary Material 1

## Data Availability

Availability of data and materialThe datasets that were used and evaluated in this study can be the corresponding author upon making a reasonable request.

## Abbreviations

HF: Heart Failure
IR: Insulin Resistance
CHD: Coronary Heart Disease
TyG: Triglyceride-Glucose
FTG: Fasting Triglyceride
HOMA-IR: Homeostasis Model Assessment-Estimated Insulin Resistance
WC: Waist Circumference
WtHR: Waist-to-height Ratio
BMI: Body Mass Index
TyG-BMI: Triglyceride Glucose-Body Mass Index
CV: Cardiovascular
EF: Ejection Fraction
HFrEF: Heart Failure With Reduced Ejection Fraction
HFmrEF: Heart Failure With Mid-Range Ejection Fraction
HFpEF: Heart Failure With Preserved Ejection Fraction
FBG: Fasting Blood Glucose
TG: Triglyceride
AF: Atrial Fibrillation
COPD: Chronic Obstructive Pulmonary Disease
SBP: Systolic Blood Pressure
DBP: Diastolic Blood Pressure
DM: Diabetes Mellitus
HbA1C: Hemoglobin A1c
CKD: Chronic Kidney Disease
eGFR: Estimated Glomerular Filtration Rate
LAD: Left Atrium Dimension
LVPWT: Left Ventricular Posterior Wall Thickness
LVEDD: Left Ventricular End-Diastolic Dimension
IVST: Interventricular Septum Thickness
LVESD: Left Ventricular End-Systolic Diameter
RVD: Right Ventricular Diameter
RAD: Right Atrial Diameter
RVFW: Right Ventricular Free Wall
IVC: Inferior Vena Cava
LVEF: Left Ventricular Ejection Fraction
LVEDV: Left Ventricular End-Diastolic Volume
LVESV: Left Ventricular End-Systolic Volume
LVFS: Left Ventricular Fraction Shortening
E/A: Early (E) Mitral Inflow Peak/Atrial (A) Filling Peak Ratio
TRV max: Maximum Tricuspid Regurgitation Velocity
AVV max: Maximum Aortic Valve Velocity
PAP: Pulmonary Artery Pressure
LVMI: Left Ventricular Mass Index
LAVI: Left Atrial Volume Index
SD: Standard Deviation
IQR: Interquartile Range
PCI: Percutaneous Coronary Intervention
CABG: Coronary Artery Bypass Grafting
ACE-I: Angiotensin-Converting Enzyme Inhibitors
ARB: Angiotensin II Receptor Blockers
TC: Total Cholesterol
LDL-C: Low-Density Lipoprotein Cholesterol
NT-proBNP: N-Terminal Pro-Brain Natriuretic Peptide
Hs-TnT: High-Sensitivity Cardiac Troponin T
RCS: Restricted Cubic Splines
HDL-C: High-Density Lipoprotein Cholesterol
OP: Obesity Paradox
NYHA-FC: New York Heart Association Functional Class
PY: Person-Year
HR: Hazard Ratio
CI: Confidence Interval
