# Supplementary material for: Association between triglyceride glucose-body mass index and long-term adverse outcomes of heart failure patients with coronary heart disease
Source: Cardiovasc Diabetol. 2024 May 9;23:162. doi: 10.1186/s12933-024-02213-2 (PMC11080126; doi:10.1186/s12933-024-02213-2)
Supplement: Supplementary file 1 — Supplementary Material 1 [file 12933_2024_2213_MOESM1_ESM.docx]

**Association between triglyceride glucose-body mass index and long-term adverse outcomes of heart failure patients with coronary heart disease**

**Contents:**

1. Figure S1. The association of the TyG-BMI index with adverse outcomes in the fully adjusted model for both diabetic and non-diabetic patients.

2. Figure S2. Association between BMI and adverse outcomes using a restricted cubic spline (RCS) regression model in all heart failure patients.

3. Figure S3. Association between the TyG index and adverse outcomes using a restricted cubic spline (RCS) regression model in all heart failure patients.

4. Figure S4. Association between FTG and adverse outcomes using a restricted cubic spline (RCS) regression model in all heart failure patients.

5. Figure S5. Association between FBG and adverse outcomes using a restricted cubic spline (RCS) regression model in all heart failure patients.

**Figure S1. The association of the TyG-BMI index with adverse outcomes in the fully adjusted model for both diabetic and non-diabetic patients**

**
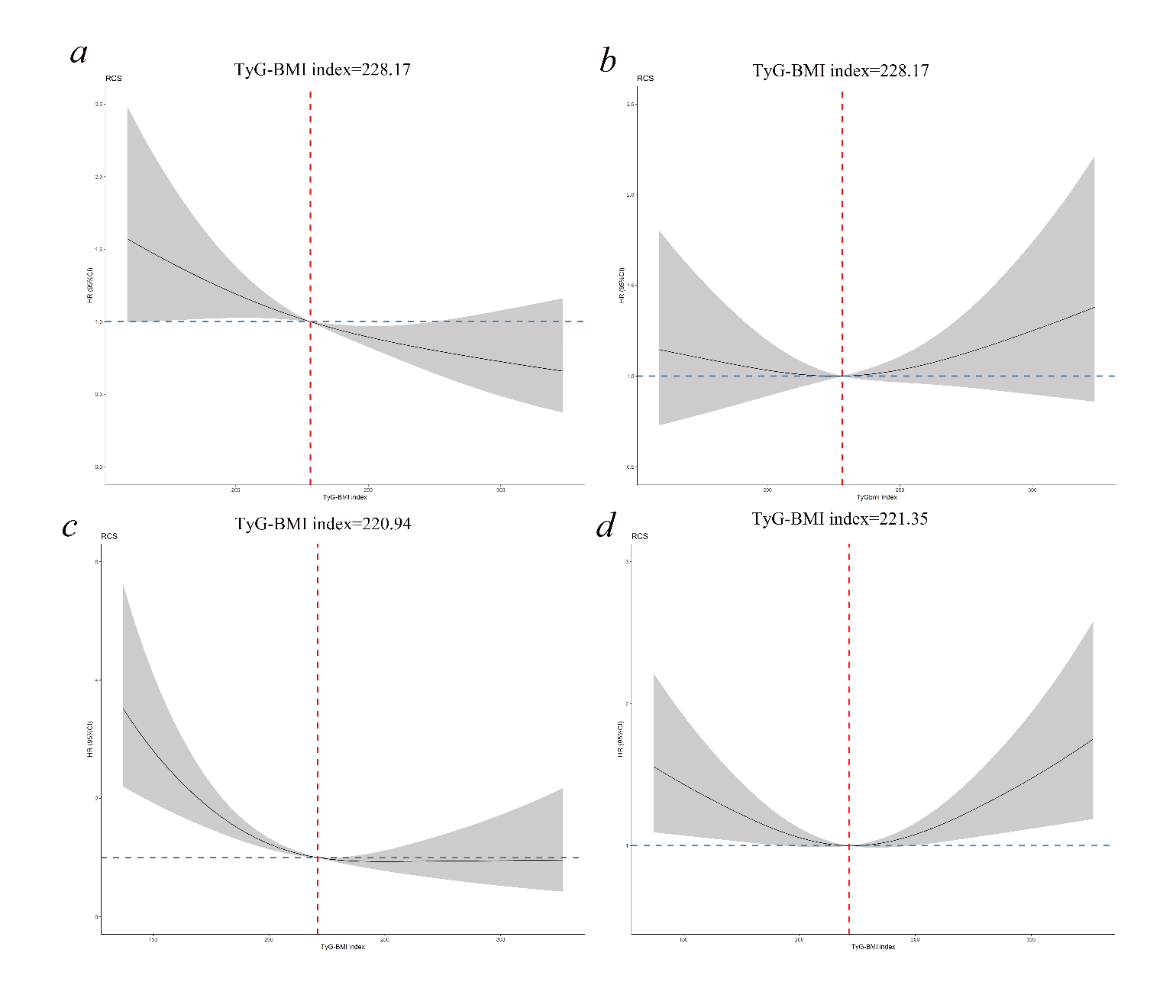
**

The association of TyG-BMI index with **a)** all-cause mortality and **b)** HF rehospitalizition in diabetic patients. The nonlinear association of TyG-BMI index with **c)** all-cause mortality and **d)** HF rehospitalizition in non-diabetic patients.

Spline curves showing the association of TyG-BMI index as a continuous variable with all-cause mortality and HF rehospitalizition in diabetic and non-diabetic subgroup. Spline curves were adjusted for age, gender, smoking, SBP, heart rate, diabetes mellitus, hypertension, previous myocardial infarction, previous PCI/CABG, stroke, chronic kidney disease, anemia, COPD, atrial fibrillation, LVEF, statin, beta blocker, ACE-I/ARB, diuretic, spironolactone, digoxin, calcium channel blocker, creatinine, TC, LDL-C, NT-proBNP, hs-TnT. When the nonlinear association is significant, the reference point is the inflection point; otherwise, it is the median of the TyG-BMI index.

HR, hazard ratio; CI, confidence interval; TyG-BMI index, triglyceride glucose-body mass index; HF, heart failure; SBP, systolic blood pressure; PCI, percutaneous coronary intervention; CABG, coronary artery bypass grafting; COPD, chronic obstructive pulmonary disease; LVEF, left ventricular ejection fraction; ACE-I, angiotensin-converting enzyme inhibitor; ARB, angiotensin II receptor blocker; TC, total cholesterol; LDL-C, low-density lipoprotein cholesterol; NT-proBNP, N-terminal pro-brain natriuretic peptide, hs-TnT, high-sensitivity cardiac troponin T.

**Figure S2. Association between BMI and adverse outcomes using a restricted cubic spline (RCS) regression model in all heart failure patients**


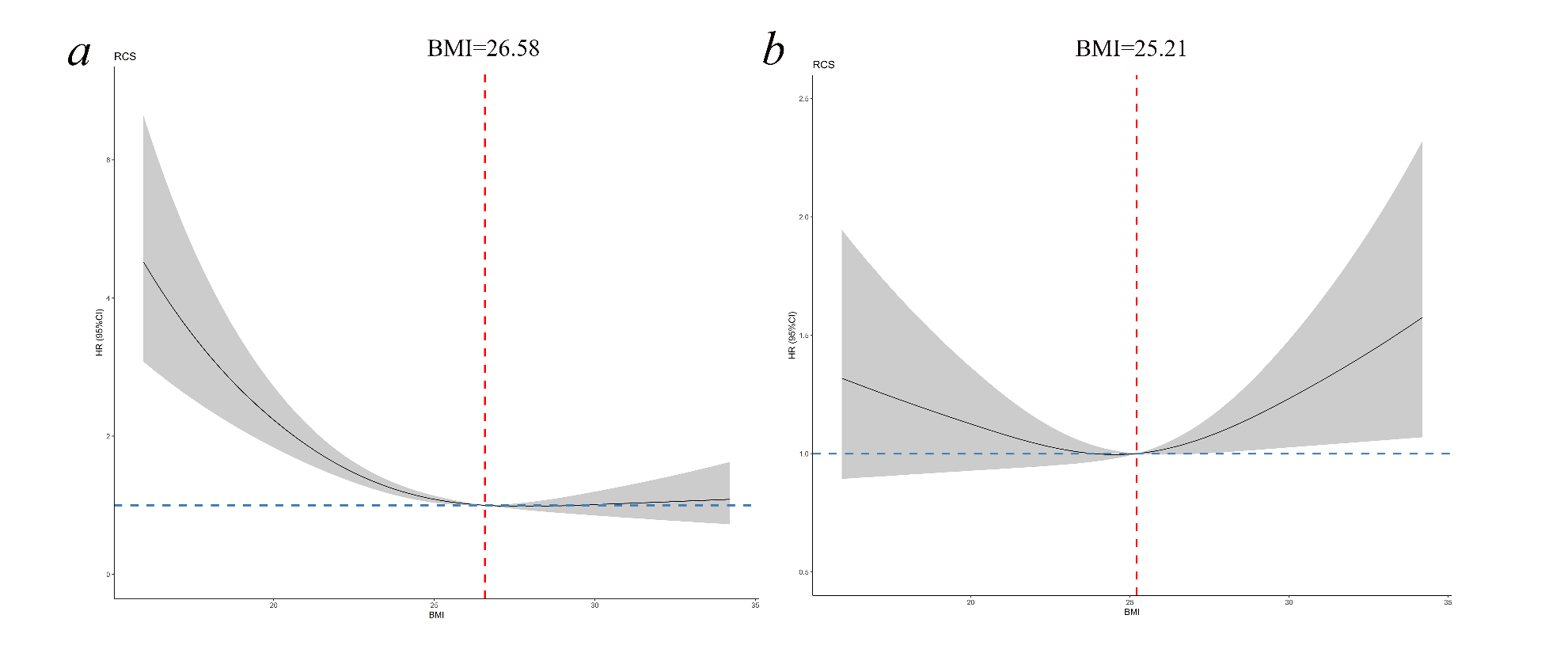


Spline curves for **a)** all-cause mortality and **b)** HF rehospitalizition.

Spline curves showing the association of BMI as a continuous variable with all-cause mortality (a) and HF rehospitalizition (b). Spline curves were adjusted for age, gender, smoking, SBP, heart rate, diabetes mellitus, hypertension, previous myocardial infarction, previous PCI/CABG, stroke, chronic kidney disease, anemia, COPD, atrial fibrillation, LVEF, statin, beta blocker, ACE-I/ARB, diuretic, spironolactone, digoxin, calcium channel blocker, creatinine, TC, LDL-C, NT-proBNP, hs-TnT. The reference point is the inflection point of BMI (**a**: nonlinear *p* = 0.001; *p* for Log-likelihood ratio < 0.001; **b**: nonlinear *p* = 0.032; *p* for Log-likelihood ratio = 0.019).

HR, hazard ratio; CI, confidence interval; HF, heart failure; SBP, systolic blood pressure; PCI, percutaneous coronary intervention; CABG, coronary artery bypass grafting; COPD, chronic obstructive pulmonary disease; LVEF, left ventricular ejection fraction; ACE-I, angiotensin-converting enzyme inhibitor; ARB, angiotensin II receptor blocker; TC, total cholesterol; LDL-C, low-density lipoprotein cholesterol; NT-proBNP, N-terminal pro-brain natriuretic peptide, hs-TnT, high-sensitivity cardiac troponin T.

**Figure S3.** **Association between the TyG index and adverse outcomes using a restricted cubic spline (RCS) regression model in all heart failure patients**

**
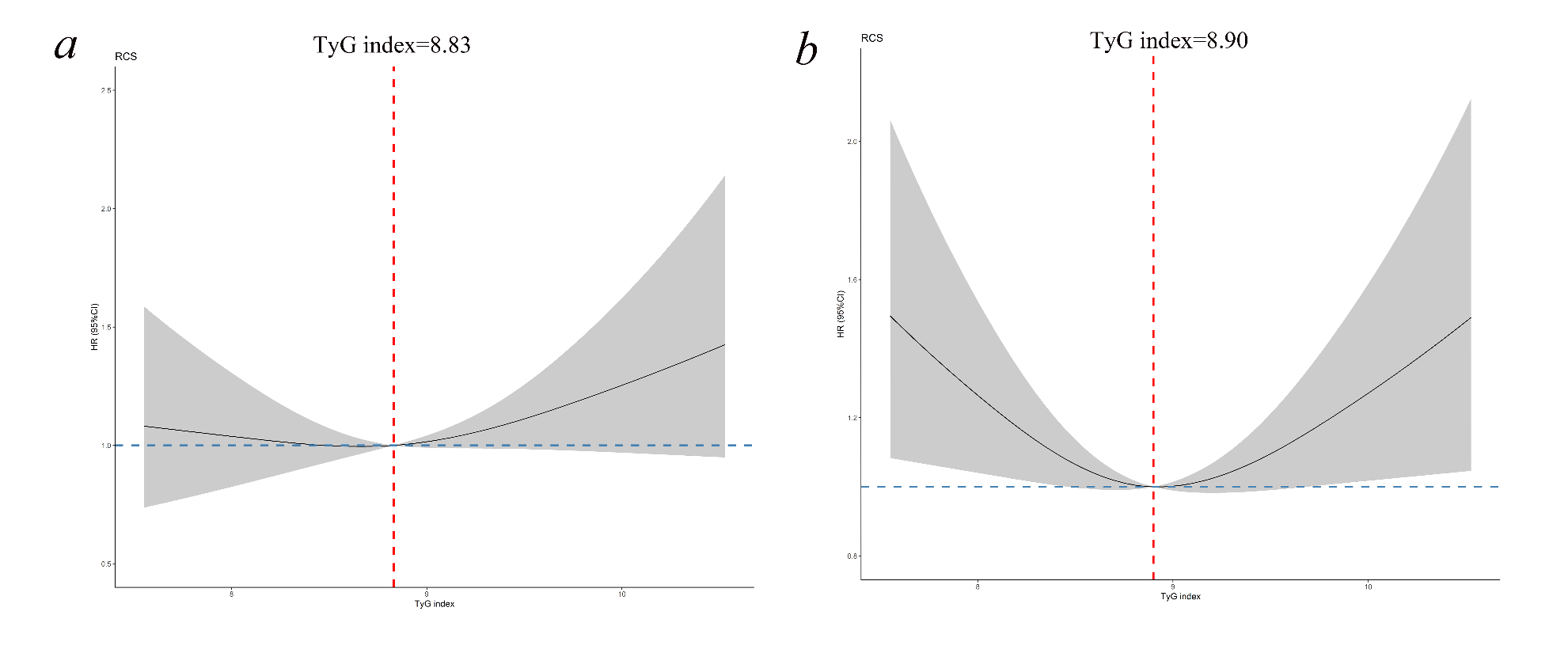
**

Spline curves for **a)** all-cause mortality and **b)** HF rehospitalizition.

Spline curves showing the association of the TyG index as a continuous variable with all-cause mortality (a) and HF rehospitalizition (b). Spline curves were adjusted for age, gender, smoking, SBP, heart rate, diabetes mellitus, hypertension, previous myocardial infarction, previous PCI/CABG, stroke, chronic kidney disease, anemia, COPD, atrial fibrillation, LVEF, statin, beta blocker, ACE-I/ARB, diuretic, spironolactone, digoxin, calcium channel blocker, creatinine, TC, LDL-C, NT-proBNP, hs-TnT. When the nonlinear association is significant, the reference point is the inflection point; otherwise, it is the median of the TyG index (**a**: nonlinear *p* = 0.918; **b**: nonlinear *p* = 0.007; *p* for Log-likelihood ratio = 0.008).

HR, hazard ratio; CI, confidence interval; TyG index, triglyceride-glucose index; HF, heart failure; SBP, systolic blood pressure; PCI, percutaneous coronary intervention; CABG, coronary artery bypass grafting; COPD, chronic obstructive pulmonary disease; LVEF, left ventricular ejection fraction; ACE-I, angiotensin-converting enzyme inhibitor; ARB, angiotensin II receptor blocker; TC, total cholesterol; LDL-C, low-density lipoprotein cholesterol; NT-proBNP, N-terminal pro-brain natriuretic peptide, hs-TnT, high-sensitivity cardiac troponin T.

**Figure S4.** **Association between FTG and adverse outcomes using a restricted cubic spline (RCS) regression model in all heart failure patients**

**
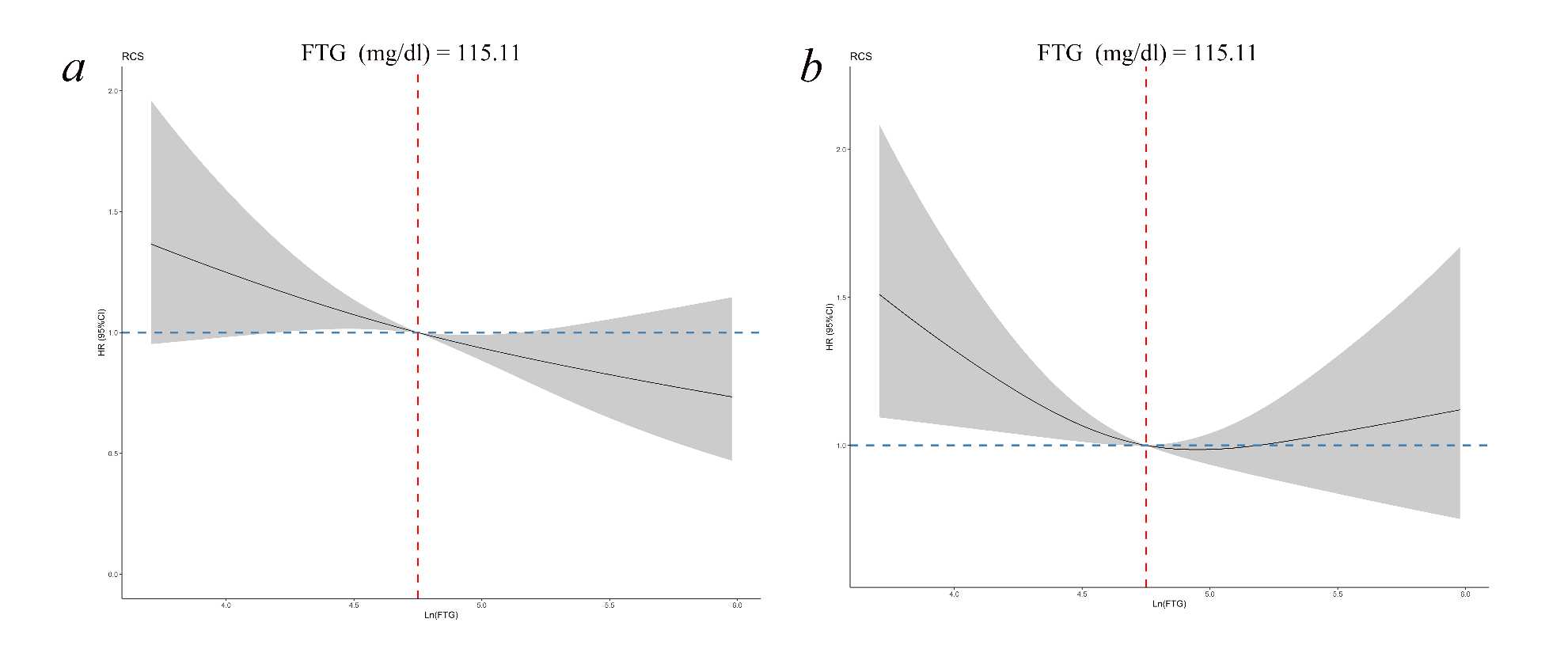
**

Spline curves for **a)** all-cause mortality and **b)** HF rehospitalizition.

Spline curves showing the association of FTG as a continuous variable with all-cause mortality (a) and HF rehospitalizition (b). Spline curves were adjusted for age, gender, smoking, SBP, heart rate, diabetes mellitus, hypertension, previous myocardial infarction, previous PCI/CABG, stroke, chronic kidney disease, anemia, COPD, atrial fibrillation, LVEF, statin, beta blocker, ACE-I/ARB, diuretic, spironolactone, digoxin, calcium channel blocker, creatinine, TC, LDL-C, NT-proBNP, hs-TnT. The reference point is the median of FTG (a: nonlinear *p* = 0.876; b: nonlinear *p* = 0.064).

FTG: Fasting triglyceride; HR, hazard ratio; CI, confidence interval; TyG index, triglyceride-glucose index; HF, heart failure; SBP, systolic blood pressure; PCI, percutaneous coronary intervention; CABG, coronary artery bypass grafting; COPD, chronic obstructive pulmonary disease; LVEF, left ventricular ejection fraction; ACE-I, angiotensin-converting enzyme inhibitor; ARB, angiotensin II receptor blocker; TC, total cholesterol; LDL-C, low-density lipoprotein cholesterol; NT-proBNP, N-terminal pro-brain natriuretic peptide, hs-TnT, high-sensitivity cardiac troponin T.

**Figure S5.** **Association between FBG and adverse outcomes using a restricted cubic spline (RCS) regression model in all heart failure patients**

**
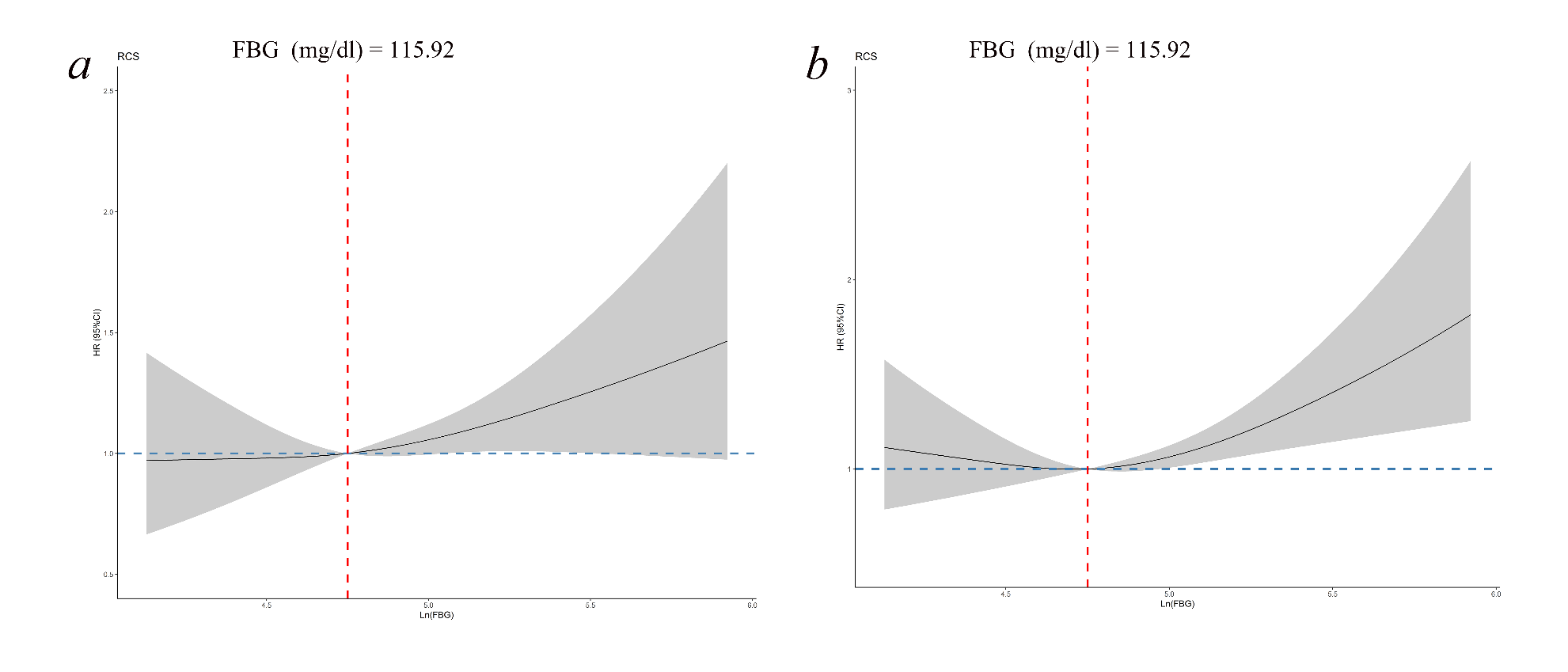
**

Spline curves for **a)** all-cause mortality and **b)** HF rehospitalizition.

Spline curves showing the association of FBG as a continuous variable with all-cause mortality (a) and HF rehospitalizition (b). Spline curves were adjusted for age, gender, smoking, SBP, heart rate, diabetes mellitus, hypertension, previous myocardial infarction, previous PCI/CABG, stroke, chronic kidney disease, anemia, COPD, atrial fibrillation, LVEF, statin, beta blocker, ACE-I/ARB, diuretic, spironolactone, digoxin, calcium channel blocker, creatinine, TC, LDL-C, NT-proBNP, hs-TnT. The reference point is the median of FBG (a: nonlinear *p* = 0.508; b: nonlinear *p* = 0.074).

FBG: Fasting blood glucose; HR, hazard ratio; CI, confidence interval; TyG index, triglyceride-glucose index; HF, heart failure; SBP, systolic blood pressure; PCI, percutaneous coronary intervention; CABG, coronary artery bypass grafting; COPD, chronic obstructive pulmonary disease; LVEF, left ventricular ejection fraction; ACE-I, angiotensin-converting enzyme inhibitor; ARB, angiotensin II receptor blocker; TC, total cholesterol; LDL-C, low-density lipoprotein cholesterol; NT-proBNP, N-terminal pro-brain natriuretic peptide, hs-TnT, high-sensitivity cardiac troponin T.
